# Experiences with cross-sectional healthcare and treatment in heart failure patients: implications for medical education

**DOI:** 10.5116/ijme.6399.eef4

**Published:** 2022-12-30

**Authors:** Anne Mette Kristiansen, Helén Lönnberg, Bo Christensen, Brian Bridal Løgstrup, Hans Eiskjær, Helle Terkildsen Maindal, Rikke Elmose Mols

**Affiliations:** 1 Department of Cardiology, Aarhus University Hospital, Denmark; 2 Unit for Teaching and Learning, Karolinska Institutet, Sweden; 3 Department of Public Health, Aarhus University, Denmark; 4 Department of Clinical Medicine, Aarhus University, Denmark

**Keywords:** Advanced heart failure, cross-sectional healthcare, multimorbidity, qualitative study, hospital-employed healthcare profes-sionals

## Abstract

**Objectives:**

to explore how cross-sectional healthcare and treatment is experienced a) by patients with advanced heart failure and multimorbidity and b) by hospital-employed healthcare professionals.

**Methods:**

Individual telephone interviews with 18 patients and close relatives were conducted. Furthermore, a focus group session was conducted with four specialised hospital-employed healthcare professionals. Purposeful sampling was used and interviews were semi-structured. Data were analysed using qualitative inductive content analysis.

**Results:**

Three main themes emerged from the interviews with patients and close relatives. These included: 1) A need for improved coordination to ensure continuity of care; 2) a plea for patient-centred care; and 3) recognition of the need to care for close relatives. Analysis of the interviews with hospital-employed healthcare professionals also produced three themes. These concerned: 1) recognition of the role and needs of close relatives; 2) limited resources for and difficulties in meeting these needs; and 3) agreement on the need for patient-centred care. Furthermore, we learned that perceived challenges are rooted in time constraints and the need for an adequate level of medical knowledge of chronic conditions and complex treatment strategies.

**Conclusions:**

This study indicates that cross-sectional healthcare and treatment of patients with advanced heart failure and multimorbidity lacked coordination, was insufficiently patient-centred and did not cater for close relatives’ needs. The study identifies patient-centredness and coordination of healthcare services targeting patients and close relatives alike as critical to proper care, medical curriculum development and continued medical training courses.

## Introduction

Worldwide, multimorbidity is part of daily life for a growing number of people.^1^ Cardiovascular disease (CVD) is prevalent in persons with multimorbidity, and more than 50% of patients with multiple chronic conditions have CVD.^2^^,^^3^ Heart failure (HF) accounts for a considerable part of individuals with CVD, and multimorbidity is highly prevalent in patients with HF and advanced HF alike.^2^^-^^5^ Various treatment strategies and guideline-based treatment pathways are available in advanced HF, including cardiac resynchronisation therapy (CRT), permanent left ventricular assist device therapy (LVAD) and heart transplantation (HTx). In patients in whom none of the above are possible, palliation is chosen.^6^^,^^7^

The rising prevalence of patients with multimorbidity challenges the highly specialised healthcare system, which is focused primarily on single diseases rather than multiple chronic conditions.^3^^-^^5^ Evidence-based management of patients with multimorbidity is known to be conflicting because guidelines and research generally focus on treating single diseases.^4^^,^^5^ Disease management with medical therapy is particularly complex, causing polypharmacy-associated risks.^4^^,^^5^^,^^8^ Consequently, patients with advanced HF and multimorbidity may undergo multiple interventions and participate in medical encounters across and within different wards, clinics and sectors, and in this way be exposed to fragmented healthcare as outpatients, in hospital and at their general practitioner (GP).^3^^-^^5^ A study supports that patients with heart failure and multimorbidity need to understand how to manage self-care within everyday life.^9^ The derived requirements on healthcare professionals need to be taken into consideration by including multimorbidity and its consequences in the curricula of medical education and ongoing training. Thus, preparing healthcare professionals by equipping them with the knowledge and skills needed to support patients with chronic diseases such as heart failure who come into contact with multiple specialties and experts is vital to providing adequate medical care. Furthermore, giving healthcare professionals the tools needed to allow patients and their relatives to achieve adequate self-management is essential.

However, patients with multimorbidity express concerns because no single healthcare professional holds the overall responsibility for their care, resulting in a lack of continuity in the healthcare services provided and absence of a patient-centred care (PCC) approach.^10^ It is now a common understanding that highly specialised healthcare systems driven by this single disease paradigm are burdensome, inefficient and ineffective in treating patients with multimorbidity.^3^^-^^7^ These issues may be further complicated by individuals with multimorbidity who find it problematic to understand health information and to interact with healthcare professionals.^11^ Actively involving patients in the medical encounter is expected to benefit the patient. Involving patients may strengthen their confidence in their own capacity to cope with their disease. Thus, in patients with heart failure, adequate patient-centred communication was shown to be associated with a reduced risk of death.^12^ Healthcare professionals therefore need to know how they can support patient learning.^13^ Teaching patients with multimorbidity how to conduct proper self-care is a part of medical professionalism^14^ that needs to be taught and developed,^12^^,^^15^ not least since more people are living with multimorbidity and healthcare systems’ financial resources are limited and should be an central part of ongoing professional evolution.

Moreover, research suggests that leadership is central to effective interdisciplinary and interagency care in multimorbidity.^4^^,^^5^^,^^16^^,^^17^ The Chronic Care Model addresses delivery of efficient health service for people with chronic illness through essential system changes intended to facilitate patient-centred and coordinated care.^18^^,^^19^ Patient-centred organisation and coordination of healthcare services for multiple cardiovascular and non-cardiovascular chronic conditions can be achieved in several pragmatic ways.^20^ Thus, optimising pragmatic approaches in cardiovascular pathways that span several different sectors may enhance health equality and create benefits.^7^^,^^20^

However, whereas much scholarly attention has been devoted to studying patterns of healthcare utilisation and care, rather less attention has been paid to patients', relatives’ and healthcare professionals’ perspectives.^21^ Thus, important gaps persist in our understanding of how patients with multimorbidity and advanced HF and hospital-employed healthcare professionals experience today’s highly specialised healthcare system, forcing patients to independently navigate healthcare systems and consult various stakeholders across and within various sectors to obtain the care needed. To bridge this gap, qualitative research is needed to explore how patients with advanced HF and multimorbidity and hospital-employed healthcare professionals experience what we understand as cross-sectional healthcare and treatment.

## Methods

### Study design and participants

This qualitative study was based on semi-structured interviews conducted within a constructivist research tradition.^22^ The aim was to explore and gain a deeper understanding of the topic of interest. In four telephone interviews, spouses participated to support the patient and add their perspectives and were thus included in the study. Data were analysed using qualitative inductive content analysis.^23^

Eligible for participation were adult patients with advanced heart failure who attended follow up of CRT, LVAD or HTx; who had one or more chronic conditions; were able to speak and understand Danish; and were treated within the heart failure clinic or ward. Hence, the included patients all had a lived experiences and knowledge about the topic of interest. Similarly, healthcare professionals employed in the heart failure clinic or ward at the hospital were eligible for inclusion. Potential participants were recruited by an experienced project nurse with no relation to the study participants. Potential participants were approached when they attended clinical follow-ups or, in the case of the healthcare professionals, when possible during their working hours. Patients with terminal heart failure or mental disorders were not invited to participate as this was considered too burdensome and risked causing discomfort and stress to vulnerable individuals. To achieve rich data, purposeful sampling^22^ was performed and we aimed for data saturation.^24^ Patients with advanced heart failure receiving different treatments, in different treatment phases and ages were recruited to ensure variation. Fifteen patients with advanced heart failure, males (n = 12) and females (n = 3), and nine healthcare professionals, male (n = 1) and females (n = 8), were sampled purposefully and invited to participate in the study. All completed a contact form allowing the first author (AMK) to contact them. Participants were then contacted one week later and the nature of the research was explained in detail again (i.e., the purpose of the study, why they had been selected, the duration of the interview). Potential participants were given opportunities to ask questions about the study, and dates for interviews were scheduled. Eighteen patients and healthcare professionals (12 males and six females) agreed to participate in the study. Additionally, four close relatives wanted to and agreed to participate in the study when the interviewer phoned the patient to conduct the telephone interview. The characteristics of the included participants are shown in Table 1.

The Danish Data Protection Agency and the Danish Patient Safety Authority approved the study. The participants also received written information about the study and the possibility of withdrawing at any time, including during the interview itself. The information underlined that opting out of the study would have no consequences for their treatment and care. Furthermore, participants were guaranteed that any individual-level information would be kept anonymous and confidential. All participants provided written informed consent was obtained (either returned in a prepaid envelope by participants interviewed by phone or handed over to the researcher onsite).

**Table 1:** Characteristics of the included participants

Patients (telephone interviews)	n
Male patients	11
Female patients	3
Years of age	35-87 (mean = 64.7)
Patients with advanced treatment	
Cardiac resynchronisation therapy CRT	6
Left ventricular assist device therapy LVAD	2
Heart transplantation HTx	6
Close relatives (telephone interviews)	
Close female relatives (spouse n = 4)	4
Close relatives to patients with advanced treatment	
Left ventricular assist device therapy LVAD	2
Heart transplantation HTx	2
Hospital-employed healthcare professionals (focus group interview)	
Occupation	
Chief physician*	1
Nurses*^'^	3
Working experience within HF	2.9–30 (mean=14.7) years

*Gender and areas of occupation not further exemplified for confidentiality reasons.

### Setting

The specific context of this study was a department of cardiology at a large university hospital in Denmark. This department is highly specialised in diagnostics and state-of-the-art treatment of every aspect of heart disease. The department cares for patients with advanced heart failure from the Central Denmark Region undergoing specialised treatment with CRT, LVAD or HTx and subsequently attend clinical follow-ups of their specialised heart treatment.

### Interviews and data collection

The semi-structured interview guide (Appendix ) was designed and based on the Chronic Care Model.^18^ The guide was pilot tested on two patients and one hospital-employed healthcare professional at different time points prior to the interviews. Minor changes were made, and the questions were otherwise found to be fit for the purpose of the study. The interviewer asked questions about the experiences of patients with advanced HF or hospital-employed healthcare professionals with experience in conducting cross-sectional healthcare and treatment, including self-management, in the context of everyday life, experiences of professional encounters and the GP’s role. The interviewer aimed for the interview to unfold as an iterative conversation between the interviewer and the participants. Furthermore, the interviewer was given a certain freedom to ask follow-up questions and to probe the interviewee by asking questions such as why do you feel this way? How strongly do you feel this? This was sought to seek elaboration or clarification of the questions in the interview guide. The probing continued until the interviewer felt that data saturation had been reached in term of obtaining a full understanding of the respondents’ perspectives.^25^ To facilitate dependability, all interviews were conducted, tape recorded and transcribed verbatim by the first author (AMK). Recordings were compared with the transcripts several times to ensure agreement. Supplementary notes were taken during and immediately after each interview to allow the researcher to document and remember important aspects that would subsequently guide reflection and analysis.^26^

### Semi-structured telephone interviews with patients and close relatives

During the COVID-19 pandemic, the study was adapted due to a ban on gathering participants in groups, which made the original study design with focus group interviews impossible, and telephone interviews were therefore completed instead. All participants participated from their home, and the interviews were conducted during weekdays from 9:00 a.m. to 8:00 p.m. Each interview started with a short repetition of the aim of this study, why the research was being conducted, the meaningfulness of the participant's participation, consent procedures and the context within which the telephone interview would unfold (e.g., format, time, reasons for recording and agenda). The participants were informed that the interviewer had no other information about them apart from their names and telephone numbers. To create a sense of connectedness and to build trust, each interview started with five minutes of informative and appreciative talking. During the interview, probing questions were asked to facilitate the interview process. Furthermore, words and tones of voice were selected carefully to reply empathically and open-mindedly to any disclosure of sensitive information. Additionally, we thought to avoid any leading questions to minimise any power imbalance. Instances of quietness played a part in the telephone interviews, triggering participants to give a more in-depth reply. Telephone speakers were switched on and interviews were recorded with a Dictaphone only after the respondent had given their permission to recording of the interview. The interviews lasted 22-55 minutes (average: 42:41 min. Total recording time: 9.9 hours of audio data).

### Focus group interview with hospital-employed healthcare professionals

The focus group interview^27^ with the four hospital-employed healthcare professionals revealed their thoughts about cross-sectional healthcare and treatment of patients with advanced HF, multimorbidity and CRT, LVAD or HTx. At the start the interview, the purpose of the study was repeated, and it was emphasised to the participants that the goal was to learn from their experiences. This was followed by various measures to create an amicable atmosphere, e.g., providing refreshments and completing an introductory round. This interview lasted 65 minutes and was conducted by AMK at the hospital facilities after working hours in a room chosen to avoid any interruptions.

### Data analysis

The participants were not asked to read the transcripts as we considered that the benefits hereof would be minimal in light of time and effort required on the participants,^28^ lack of response or responses without any feedback,^29^ and because participants had expected and agreed on just one encounter and had been promised anonymity by the interviewer. Instead, the interviewer rephrased, asked for clarifications several times during the interviews to confirm what participants meant and encouraged them to support their statements by providing examples. This was done to empower participants and seek immediate feedback on the initial understanding, while participants had a clear recollection of the questions and conversation and were still able to recognise themselves or their particular experiences. This allowed participants to correct details and elaborate even further on their reasons and experiences.

We used a qualitative content analysis to analyse data following the process described by Graneheim and Lundman.^23^ Thus, the focus was either on the manifest content (i.e., the participants' descriptions) or on the latent content (the underlying interpreted meaning of descriptions). The material was read and re-read several times to gain an in-depth understanding. Subsequently, an inductive content analysis was conducted. First, meaning units were formed, condensed and abstracted into categories, i.e., we analysed what was said in the telephone interviews or during the focus group interview (the manifest content) in relation to cross-sectional healthcare and treatment. Next, we focused on understanding the latent content of the categories and formulated these into themes. Table 2 and Table 3 illustrate the analysis. The primary analysis was performed by the first author guided by the research question and was followed by a triangulation of different parts of the analysis conducted by three of the authors (AMK, RM, HL) to ensure coherence and consistency of the findings. These three authors (all researchers) held regular meetings during the process of analysis to critically review and repeat conversations to establish which statements would fit into the emerging categories and themes, and to identify any details that the first author might have missed or misinterpreted. This process continued until an agreement was reached. Notes from the interviews were assessed and used as supplementary data and included aspects that participants had stressed or questioned during the interviews. These notes also included the researcher's reflections on the interview. Data collection and analysis phases were conducted in parallel, allowing any thematic categories identified in the early transcripts to be explored in the subsequent interviews. All quotations were translated from Danish into English by a person who is proficient in English, and three of the authors (AM, RM, HL) reviewed and checked that the translation of the quotations was meaningful and in agreement with the original data.

### Trustworthiness

We used Graneheim and Lundman^23^ to assess trustworthiness. To ensure credibility,^23^ we described our research process carefully and we used methodological triangulation by gathering data using relevant data sources and different methods such as telephone interviews, focus group interview and supplementary notes and by taking various perspectives into account. We used investigation triangulation by involving members of the research team (AM, HL, RM) in the process of analysis; each of whom first conducted a separate part of the analysis, which was followed by discussion of the participants’ responses. To illustrate, we have provided examples of the analysis (tables) and reproduced citations herein. Data collection was an evolving process as the second telephone interview was slightly different from the first interview in terms of the wording of a few questions. Thus, overall, the second interview was guided by the same questions as the first interview to meet the requirement for dependability. We further sought to provide a good description of the participants, the sample strategy and inclusion and exclusion criteria; and we have described the context and setting of the research, the theoretical foundation for the research, the interview procedure and topics, added the interview guide and presented our findings with supporting citations to allow others to appraise these data and mirror them to other contexts to meet the transferability criterion. Furthermore, we reflected thoroughly on potential researcher bias as we are aware that in qualitative research, the researcher plays an active part and may therefore potentially affect the results.^30^ In this study, researchers were experienced nurses, chief physicians specialised in cardiology and another healthcare professional specialised in educational research. Therefore, a critical perspective and probity were upheld by constant self-reflection and self-questioning during the process of collecting, analysing and interpreting the data to ensure that these elements were valid and based on data.

**Table 2:** Examples from the content analysis process; condensed meaning units, categories and sub-themes corresponding to the three themes from interviews with patients and the four close relatives

Meaning units (Examples of each theme)	Category	Sub-theme	Theme
“To begin with I got a high dose of a medication named Sotalol. I got so sick from taking them. I asked and got permission from the doctor to reduce to half the dose. But it is risky to do it he said, you need to know that, and it is YOUR risk. I will write that you can do it if you want and that you have pressured us to get permission. But I am happy because I got a completely different life when the dose was reduced. I had some ping-pong with the doctor and I needed to argue why I wanted this. There were no angry response or finger-wagging – except from the one doctor who said: "then you might as well refrain from taking anything because then it does not work". However, in my case this was not true because even a pain killer affects me heavily…" (Male, CRT, R16)	Experiencing side-effects means that patients take part in their treatment. A need to argue, negotiate and take responsibility for adjustment of treatment is experienced	Involved in decision-making	Interacting within a contemporary healthcare system
“The pacemaker has given me 10 years which I otherwise wouldn’t have had. I have one that can revive me an OCD [ICD] or what it is called... I can't remember. It has never been used, but they can see that on the damn telephone [round-the-clock monitoring] that we have lying beside the bed. I have always thought it is pretty expensive stuff just lying there, if it is not doing anything.” (Male, CRT, R13)	Thoughts about the heart condition and understanding what a medical device does.	Learning and adapting to a new life situation	Aiming to continue a normal life in the context of the complexity of disease, treatment and self-management
“I think that when you have an LVAD, it also interferes with the life of the close relatives. I do not know if it interferes more with my wife’s life than that of others because I need to have a reminder that I need to change the battery and all that. It may also affect their lives so what do they think about it? People always ask me what I think but they never ask her how she feels about it.” (Male, LVAD, R3)	Thoughts about how the condition and treatment affect family members who no-one asks about	Close relatives receive little attention and support from professionals	Lack of support for close relatives

**Table 3:** Examples from the content analysis process; condensed meaning units, categories and sub-themes corresponding to the three themes from the focus group interview with hospital-employed healthcare professionals

Meaning units (Examples of each theme)	Category	Subtheme	Theme
“I think we try and help coordinate if a patient comes and says “I am going in for a check this day and that day´. If it is easy then we help, but we will not move heaven and earth to make it happen.” (Female, nurse, R19)	Trying to help coordinating appointments when patients request it, provided it is easy and not too time-consuming	Acknowledging a lack of coordination within the healthcare system	Operating in a contemporary healthcare system
“They can be very scared and anxious. We experience that when they call four times a week until they get back on their feet. They can have all kinds of questions.” (Female, nurse, R21)	Anxiety and doubts as a driver for patient questions and contact to healthcare professionals. The need to learn in a new life situation with HF	Accessibility of healthcare professionals serves to overcome patient doubts and worries	Understanding patients’ need for support and expressing doubts and queries
“We invite close relatives because there are so many practical things like changing bandages, alarms and how to react, how to change a battery and how to change the controller. That is why we invite them. We are often in dialogue with them. It is very often the close relatives who phone and say “we” have a fever but other close relatives we rarely see.” (Female, nurse, R21)	Healthcare professionals take initiatives to involve close relatives to assist with practical patient care and experience that the involvement of close relatives is very diverse	Taking on the role of care provider and care coordinator	Acknowledging the role and engagement of close relatives

## Findings

### Cross-sectional healthcare and treatment experienced by patients and close relatives

The analysis of the interviews with patients and close relatives about their experiences with cross-sectional healthcare and treatment revealed three main themes. The themes “Interacting within a contemporary healthcare system” and “Aiming to continue a normal life in the context of the complexity of disease, treatment and self-management” elucidated the role and actions of patients and close relatives to navigate within the healthcare system and participate in professional encounters while dealing with self-management in the context of everyday life with heart failure and multimorbidity. The third theme “Lack of support for close relatives” was a recurrent and prominent issue across the entire data material and revealed that close relatives are not involved in or supported by all parts of the system despite their active role, interest and engagement in and commitment to the patient treatment and care. All themes are described separately below and illustrated with quotations.

### Interacting within a contemporary healthcare system

This theme focused on how a HF diagnosis forced patients and close relatives to manage diverse and complex treatment and self-management-related challenges within a highly specialised healthcare system.

The theme clearly revealed how clear coordination lacked and relied on the ability of patients and close relatives to assume responsibility for the process. Patients experienced being supported, but their close relatives were offered no support.

### Attentive and professional meetings within cross-sectional healthcare

Most patients found that attentive and professional meetings were characterised by healthcare professionals being friendly, showing a surplus of mental resources, taking time to provide the information needed and paying attention to their concerns. This made patients feel that they mattered as individuals.

Other patients emphasised that healthcare professionals’ approach reduced their anxiety and helped them endure the situation when healthcare professionals signalled that they had time for them and provided physical comfort. However, some patients described that their treatment at times felt impersonal and this made them feel that they were no more than a piece in a puzzle. Patients perceived that this was due to a need to limit costs and for doctors to gain experience.

"They actually had amazingly good time, but you are part of an assembly line. You are put on a shelf on the assembly line and go forth as the next in line. It's not so nice to think about, but that is the way you can operate reasonably cheaply today and the way the doctors become skilled". (Male, CRT, R16)

Generally, patients and close relatives perceived that the treatment and care provided by healthcare professionals within cross-sectional healthcare was meaningful and provided in a competent manner, promoting a feeling of safety. However, a few felt that the healthcare professionals did not take their complaints seriously when obvious doubts were displayed in situations in which the patients’ previous understanding and experiences fell short. This made some patients feel abandoned and made them reluctant to seek help from healthcare professionals when they had doubts.

"One example could be when I take a picture if I see a red circle around the area where the lead comes out and then call them and tell them that I think something is wrong. When I then go to get it checked at the hospital and the doctors just say, "that is what we're expecting". Then I sometimes get the feeling that they just see me as hypochondriac who comes to the hospital for no good reason.” (Male, LVAD, R3, patient)

Few patients were able to recall that healthcare professionals had asked about their needs and experiences during their hospital stay, and some patients expressed that being at the hospital might have limited their ability to listen to questions. Other patients described loneliness and reluctance to disturb when healthcare professionals did not inquire about their needs and experiences because they were busy. Similarly, when nurses took time to talk with patients, it made the patients feel remorse.

"No, I don't think they ask, and it feels like you are left on your own. The nurses do not just come and sit with you. They have a lot to do. Damm, they run fast. I think they could easily use more hands. The part of their job which is talking to patients takes time from something else, so when you sit down and talk to them you almost feel guilty about it". (Female, HTx, R18)

### Involved in decision-making

The patients expressed being involved in multifaceted crucial choices about treatment, e.g., yes/no to LVAD, taking medication or not, when and if it is time for follow-up and more general self-management decisions. The patients described that they felt involved when information was conveyed and when decisions were discussed and understood, and when agreements were made. Occasionally, this involved patients negotiating and taking responsibility for changes in their treatment. Other patients described that they received the best treatment as decided by the healthcare professionals and expressed that they lacked the knowledge to take part in any decision-making or the ability to ask questions. A few patients perceived medical treatment as the medical team's responsibility and described this as a reason for their lack of involvement and for not taking part in the decision-making.

"I do not know how it is supposed to be. I have faith in authority, so I listen to what they say and do what they want me to do. I do not really ask questions. Because they know better than I do. I can feel that I get better after following what they have said, so it cannot be all that bad…" (Male, CRT, R13)

### Trusting and understanding information

According to the patients, information within cross-sectional healthcare was trusted and mainly conveyed deliberately by both doctors and nurses and concerned the patient’s condition, plans for treatment, ways to support self-management, examinations, functions of e.g., the CRT system, etc. However, use of medical language was a barrier to patients' understanding, making it hard for them and their close relatives to understand parts of the information. Understanding was facilitated by relying on support from close relatives, showing persistence in seeking clarification or years of experience as a patient.

“In the beginning, it was medical information we received, and I am an upholsterer and my wife she is a child-and-youth-worker so we did not understand a word of what they said. As we became more experienced in the hospital system and after the hospital started to be good at informing differently, then it helped a lot. Today, we know some of the “fancy words” [Latin] and know what they mean. It makes things a little easier and we are not afraid to ask. Not even 2 or 3 times if we do not understand the answer. Today, you must ask because otherwise you do not get enough information. If you don't ask, people will think that you do not want to know.” (Male, CRT, R16)

### Patients take the initiative to coordinate with support from close relatives

Only patients with a long travel time described well-coordinated follow-up visits. All other patients reported that coordination of follow-up of chronic diseases was inadequate and did not automatically occur, causing some patients to have several separate appointments within a single week. Participants in this study expressed this as frustrating, and it made patients and close relatives take responsibility for improving the coordination and assume the role as care coordinators, linking together different departments and clinics. Despite these efforts, worries about appointments coinciding were expressed and when this occurred, patients described that they decided which appointments they would need to cancel.

“If there are months where I need to go for 2 or 3 things, then we call them and ask if is possible to do it all in one day; for instance, the skin department where I also go for check-ups. Sometimes it is possible and sometimes it's not.” (Male, HTx, R5)

Notebooks, paper or telephone calendars were formats used for self-management by patients and close relatives as they themselves tried to build an overview of appointments and follow-ups.

“It can almost be impossible to keep track. I need to write it all down in a notebook, which I look through each week to see where I am with my appointments. Then I have my wife and she is very good at keeping track. That is really good. But it is a problem to keep track. We try to put the puzzle together. There is no automatic way of doing this at any of the hospitals. They hardly know that I go for check-ups elsewhere and not even if I refer to it and say it every single time. They only check up on this if I force them to”. (Male, CRT, R16)

### Improving the healthcare system in the patients’ eyes

Patients wished for better coordination of appointments between and within hospitals for follow-up visits, opportunities for in-hospital conversations with a specialised psychologist with knowledge of HTx for the transplanted patients and their close relatives, opportunities to speak with other patients with a lived experience of HTx, continuity with respect to healthcare professionals, co-management of extra check-ups, tailored rehabilitation after HTx and would have liked to be able to order hospital medication online.

“The only thing we would like is if you could coordinate it [hospital appointments] a bit better. For instance, if we have 3 visits within the next 14 days. Perhaps you could put the two of them together or all 3. We do not care if we have to meet at 7:30 in the morning and then stay the rest of the day. (Male, HTx, R5)

All patients and close relatives pointed to a lack of support for close relatives, including support for children with a severely ill parent. A lack of this kind of support was described to have a long-lasting, negative effect. Here, patients described how they were surprised to learn that self-help groups for children with parents with a heart disease did not exist but were established only for children to patients affected by cancer or mental illness. This led some patients to pay for psychologists themselves. Therefore, patients suggested making groups for children to parents with heart diseases and establishing teams caring only for close relatives. It was stressed that healthcare professionals should signal that they have the time, acknowledge when close relatives are present and pay attention to their needs and feelings. However, it should be kept in mind that close relatives are concerned about taking focus away from the patient and aware that their need for support depends on the patient’s situation.

“You should probably pay more attention to close relatives. Because I think they are forgotten. If I had not said anything, then she would not have been involved and I think that is a big problem that close relatives experience. They keep it all to themselves. There is no one to talk to. I do not know how easy it is because healthcare professionals may not have time, and, of course, doctors need to focus on those who have the medical problem, but maybe you could establish a kind of a team to deal with close relatives. I think that could be a really good thing. If you are the close relative of a patient with cancer, you get all kinds of support - also the children. There [in the field of heart disease] you don't have any support for the children or for close relatives.” (Male, HTx, R10)

This patient described one occasion during which healthcare professionals paid attention to his wife.

“The doctor looked at my wife and said: `How do YOU feel? ´ She completely broke down. No one had asked her that before. I will never forget it. I have never seen my wife like that at all. The doctor and nurse, they spent a whole hour talking to her. It was amazing." (Male, HTx, R10)

### Different roles of and expectations to general practitioners

Patients’ and close relatives’ experiences with GPs were described as positive when the GP showed an interest and took the time needed to listen to and discuss issues and possible solutions. It was also positive if the GP communicated with and stayed well informed about progress in the patient’s treatment made elsewhere in the healthcare system. Furthermore, it was assessed as positive when the GP respected former medical decisions, sought advice and support from peers and offered psychological support. If patients and relatives did not experience these elements, they felt alienated and only rarely consulted their GP.

“My GP had previously been a cardiologist, so I thought he must just be the doctor for me. He is extremely unpopular, but I think he is great. He was probably not attending when they learned about empathy in medical school, but, fortunately, I am the type who wants to know things directly. He always says, `listen to me, I may be an old cardiologist, but it is the specialists at the hospital who manage your medication. If I have a suggestion, you should call them – your lifeline´. He has said from the beginning that he does not take a stand on anything. It should be the hospital that says yes or no. I'm being asked to call while I'm sitting in his consultation. He is a tough one, you know?” (Male, HTx, R10)

### Aiming to continue a normal life in the context of the complexity of disease, treatment and self-management

This theme highlights that dealing with advanced HF, multimorbidity and treatment within cross-sectional healthcare was closely intertwined with the patients’ and their close relatives’ everyday life and could not be considered separately. The drive for self-management in this context is carried by patients’ desire to enhance their learning and self-directedness and to gain information about bandaging, taking medication, using advanced technology as part of their treatment and knowing who to turn to for help and guidance. Patients' prior knowledge, beliefs and experiences influenced their understanding of their current situation.

### Perception and understanding of disease and multimorbidity in daily life

Some patients were affected by and expressed feelings of hopelessness about their situation and life as they had not understood their treatment plans or the rationale guiding these plans; others did not describe themselves as ill, albeit multimorbidity requiring that they performed multiple self-management tasks every day. Their multiple chronic diseases were either understood as a natural consequence of their HF trajectory or patients mentally suppressed their situation. Some patients did not question things but explained that healthcare professionals’ lack of attention towards other chronic conditions showed that they lacked medical knowledge. Several patients described that many years of experience with diseases and prior knowledge had formed their pre-understandings, which they used to make sense of any given situation.

“I might be a strange person because in my own mind, I do not see myself as sick because I feel fine and I don't even feel like I am taking any medication, but I do.” (Female, CRT, R15)

### Learning and adapting to a new life situation

Several patients expressed how their HF diagnosis, their comorbidity status and the complex treatment they received had prepared and motivated them to learn how to manage the situation and to, e.g., understand and use new technologies. Another driver of patient learning was a desire to change how situations were dealt with over time due to their HF trajectory. However, this troublesome learning process was rooted in questions and doubts, and it was affected by the patients' pre-understandings. Constant, around-the-clock access to healthcare professionals helped patients to understand and interpret bodily sensations and worries while reducing anxiety and stress in both patients and close relatives.

“The opportunity to call healthcare professionals 24 hours a day means everything. If I wake up at night and I do not feel well, then I can call them. In the beginning I was a bit reluctant to call, but then I learned it was okay. I think it was a nurse from the ward who said `listen, it is better you call us right away and then we can tell you if it is something you need to worry about. If you need to go to the hospital or not. Then you won't have to think about it any longer’. When, as a patient, you have managed to learn this, then this opportunity is really good.” (Male, HTx, R10)

### Lack of support for close relatives

Dealing with advanced HF and comorbidities within cross-sectional healthcare affects patients’ close relatives and patients’ everyday life. Despite this, patients described how they conveyed information about treatment and self-management behaviours to their close relatives instead of healthcare professionals. They also described that close relatives, on whom several of the patients relied for daily support to follow treatment plans and carry out self-management, participated in follow-ups at their own initiative. Both patients and all close relatives commented on the failure of the system to provide support for close relatives in cross-sectional healthcare.

### Lack of involvement when information is conveyed

Some patients explained that the information they conveyed to close relatives concerned advanced treatment such as getting a pacemaker and around-the-clock monitoring.

“My wife has only been informed to a very limited extent. The information she received about the pacemaker she got from me. I have also told her about the around-the-clock monitoring.” (Male, CRT, R11)

### Close relatives receive little attention and support from healthcare professionals

Both patients and close relatives expressed that healthcare professionals paid too little attention to the close relatives’ needs and feelings and that this would be a barrier to detecting relatives' concealed emotions regarding the patient's situation.

“Healthcare professionals could be a bit better at asking how the close relatives feel. I have needed that sometimes. You could consider this in the future. Just go in and ask the close relatives. I actually think that it will mean a lot. So, that you, as a close relative, can also express what you think and how you feel. I know that the patient is more important, but the close relatives could also have some issues they carry with them and need to discuss. Healthcare professionals could maybe be those you offload your worries to. I am serious and I really mean it because I have years of experience, and no one has ever asked me: `How do you feel about all this´. I could have wished for that sometimes; especially when he got the LVAD. Then maybe I could have sat down and said: `I feel like this and that´ and what should I do to pull through. I have just been keeping it to myself.” (Female, spouse to patient with LVAD, R4)

### Deep involvement in managing patient treatment and care

In this study, close relatives’ involvement and participation in the patients' treatment and self-management meant learning how to change bandages and providing practical assistance, shifting batteries, making clinical observations and administering medication. Close relatives felt that this was natural and a way to support the patient’s self-management.

“It sounds easy and I think it was the intention that it should be easy. But it is not that easy. I need to pay attention to how he feels. Because I can see it in his face if he turns ill. I need to pay attention because the LVAD is not always working according to plan. For instance, when it writes “low flow”, then I need to pay attention because his face may turn grey – so I need to keep an eye on him; `Will something happen, or will it pass´? I am the one who changes the batteries when he doesn’t. I change the bandages. I have learned that because they have taught me how.” (Female, spouse to patient with LVAD, R4)

All patients and close relatives in this study described how close relatives supported the patient by being present at clinical follow-ups on their own initiative and not because they had been invited by healthcare professionals. Patients wished for their close relatives to participate, regardless of their disease trajectory.

“My wife, she says that she wants to participate. She says that I miss too much of the information. When we both participate, we obtain a bit more information. Once I asked if she could participate and they said yes. Now, she always participates.” (Male, CRT, R13)

### Cross-sectional healthcare and treatment experienced by hospital-employed healthcare professionals

The three main themes found in the focus group interview with the hospital-employed healthcare professionals were overall similar to the themes found in the telephone interviews with patients and their close relatives. The theme “Operating in a contemporary healthcare system" elucidated the possibilities and challenges associated with professional encounters. The themes “Understanding patients' need for support and expressing doubts and queries" and “Acknowledging the role and engagement of close relatives" uncovered aspects that were found to affect hospital-employed healthcare professionals' approaches to and experiences with taking part in the treatment and care of patients with heart failure and multimorbidity.

### Operating in a contemporary healthcare system

The healthcare professionals in our study described that they felt supported in their professional roles when clinical pathways and supporting tools were available to ensure quality and equality in the treatment and care of patients with advanced HF. Treating patients with a chronic disease for years was described as a process that meant creating a professional relationship and which made it easier to control treatment. However, some expressed that it was challenging to build an overview of several chronic conditions in addition to HF, while others did not mention these if the patient’s condition was stable. The need for improved coordination in the healthcare system was stressed, with patients taking on an active role.

### Clear patient pathways and a sense of knowing and control

Closely scheduled follow-up of patients undergoing HTx was described as enabling healthcare professionals to detect and manage problems related to the patient's treatment and care. Ensuring consistency in healthcare contacts was explained as system changes aiming to make patients feel safe and acknowledged.

“The continuity of care for patients undergoing a heart transplant is fairly good, I think. If they get past the transplant and everything is going according to plan, we meet them on a weekly basis, which I think is great as this allows us to keep them “on a short leash”. If problems arise, we can act on them fast.” (Female, nurse, R20).

### Grasping the big picture and knowing enough can be challenging

Some healthcare professionals did not inquire about the patient’s other chronic conditions when the patients were in a stable phase and the chronic conditions did not interfere with the treatment. Others described it as difficult to build an overview and have enough knowledge about other conditions and expressed that having an adequate amount of knowledge of the heart disease and its complex treatment could be challenging in its own right. Furthermore, they believed that patients' prior knowledge, beliefs and understandings were very important for the collaboration about treatment and self-management in cross-sectional healthcare.

 “If the patient has an uncomplicated diabetes, I may not go so much into that. I do not know so much about that. But if something affects the treatment or interacts with the medication and the treatment we are about to give, then I hope that I will pay attention to it.” (Male, chief physician, R22)

### Acknowledging a lack of coordination within the healthcare system

The healthcare professionals thought that coordination of follow-up could be optimised, e.g., supported by technological solutions, but also that it was the patient's own responsibility to make changes as needed. Some expressed that they failed to manage coordination due to a lack of time. Only few paid attention to future appointments elsewhere in the healthcare system. This inattention was acknowledged as putting an unreasonable responsibility on the patients and being counterproductive.

“The other day, I had a patient who has a fulltime job and on top of this he has several chronic diseases and that is also a fulltime job because he had to go to a lot of clinical follow-ups, so he could hardly take care of his real job. Then he actually said no to treatment with anticoagulants because he simply couldn't manage it. He opted out because he couldn't allocate the time for it.” (Male, chief physician, R22)

Health professionals described collaboration with the patient’s GP as non-existing due to longer periods of follow-up at the hospital of patients with HF, and because they had the impression that GPs nowadays are responsible for fewer things than previously due to organisational changes in Danish healthcare.

“I would say that the patient's GP is almost by-passed because patients are followed much longer at the hospital due to their heart disease. However, when the follow-ups end, then the GP becomes important. Nowadays, the GP wouldn't even take any blood samples or write a prescription. They do so less and less. So, no there is almost no collaboration with them.” (Male, chief physician, R22)

### Involving patients by conveying individually structured information

The healthcare professionals described that patient involvement occurred when they conveyed information about HTx, other treatment decisions, symptom recognition and reactions to these and medication, and when they trained patients in practical self-management skills to underpin their understanding of and responsibility for the treatment.

“We almost have a scheduled programme and checklists of what we need to go through for patients who are transplanted. Sometimes you need to make this individual. It is almost the only group of patients we care so much about informing about medication at this point. They sit and dose the medication themselves because they need to know what they put in their mouths and what the pills look like. We also insist that they can count the pills themselves to know how many they have when they come for a biopsy again.” (Female, nurse, R19)

### Understanding patients' need for support, expressing doubts and queries

All healthcare professionals in this study understood patients’ and close relatives’ need to have a "life-line" with 24-7 access to help and support. They expressed, however, that other ways of providing support were limited.

### Accessibility of staff overcomes doubts and worries

The healthcare professionals found it difficult to offer adequate support for patients with few resources, but described that when patients or close relatives called, the call allowed them to attend to queries for and provide timely feedback, and they expressed that this was a way to help reduce anxiety in situations and to underpin self-management.

 “I think it is a very good idea to have that telephone because it gives patients the opportunity to get a quick answer to their questions. Recently, I had a patient where the ultrasound of the heart had shown that some parts of the newly transplanted heart were probably a bit thicker. As professionals, we were not so worried and suggested to wait and see. But for the patient, this was quite a big deal. He was told to wait for a new ultrasound of the heart. We can just go home; but for patients, it is their reality all the time, so the opportunity that they can call if they are worried is good and makes sense.” (Female, nurse, R19)

### Acknowledging the role and engagement of close relatives

This theme highlights that the role and involvement of close relatives differs but depends mainly on the patient's motivation, the wish and caring nature of the close relatives, the patients' needs for practical support and negotiated roles in the patient-relative relationship. The healthcare professionals

described that close relatives’ involvement was on their initiative and that patients who were supported had a better illness pathway. They acknowledged, though, that the close relatives’ roles could be stressful and lacked professional attention.

### Taking on the role as care provider and care coordinator

The healthcare professionals described that they involved and relied on close relatives to help the patient manage practical issues in relation to advanced treatment at home.

“I think we try to recommend that relatives participate in all parts of the care and in all appointments.” (Female, nurse, R19)

“I think it is very important that a patient with an LVAD has a relative because they have this machine at home and there might be alarms that need to be handled and bandages that need to be changed. So, it is important with social back-up if you can call it that.” (Male, chief physician, R22)

### Need for more focus on close relatives

The healthcare professionals found it challenging to adequately support close relatives due to a lack of time, but they recognised the stressful nature of being a close relative of a patient.

“I think having a focus on close relatives to critically ill patients is not always easy and it is a big task. Sometimes, I think they [the relatives] can be forgotten because we are too busy. I think we can improve this. But then again how much can we offer to help them?" (Female, nurse, R19)

## Discussion

The aim of this study was to illuminate patients and hospital-employed healthcare professionals experience with cross-sectional healthcare and treatment for patients with HF and multimorbidity.

Providing coordinated and supportive care within cross-sectional healthcare of patients with multimorbidity and their close relatives would be in agreement with a patient-centred care approach^31^ and the Chronic Care Model.^18^ Similar to other studies,^8^^,^^20^ this study showed that patients with advanced HF and multimorbidity and their close relatives experienced that they must navigate within a highly specialised healthcare system. Both patients and relatives experienced a fragmented healthcare system that was inadequately organised to support patients with multimorbidity. They faced appointments at different locations, and the healthcare system primarily focused on one condition at any time. Previous research has yielded similar results.^32^^,^^33^ In the present study, these experiences were reported as frustrating and they may constitute a risk to patient safety if issues impacting on treatment are overlooked.^33^ As also reported by others,^34^^,^^35^ we found that a clear lack of coordination within cross-sectional healthcare generated an even greater burden and placed an even higher responsibility on patients and close relatives who need to take on the role as care coordinators. This highlights several concerns. First, lacking coordination affects the care provided for and also the quality of life of patients with HF and multimorbidity.^32^^,^^33^ These results resonate with our findings describing how lack of coordination between locations and appointments may make patients opt out of relevant treatments and decide themselves which appointments to prioritise. This may be perceived as contradictory to evidence-based HF care^7^ and suggests that proactive scheduling of appointments would impact positively on patients' possibilities to manage their job and family life and also help avoid that appointments coincide. According to the healthcare professionals of the present study, a lack of coordination within cross-sectional healthcare was attributed to inadequate IT systems, a lack of time and high workloads. Second, in the present study, close relatives played a crucial role in keeping track of healthcare appointments. Our study also showed that - despite their important role – close relatives did not feel invited to take part in the patient's treatment and care. Yet, they had various approaches to self-management by which they aimed to help the patient navigate the system, maintain an overview of appointments and gain a sense of control.

The present study showed that both patients and close relatives were forced to learn how to navigate within cross-sectional healthcare, which included making decisions and taking action to achieve better coordination and following and carrying out complex treatment. We argue that their learning should be explicitly acknowledged as a part of cross-sectional healthcare as learning cannot be separated from the disease course; patient learning is an integral element in supporting patients' self-management. Patients with LVAD need support from relatives.^20^^, ^^36^ This study showed the extent of the responsibility of close relatives supporting, e.g., patients with LVAD. Close relatives support included observation and assessment of, e.g., clinical symptoms and taking action to prevent worsening of the disease. The responsibility experienced by close relatives is likely to have a negative psychological impact and may possibly also negatively affect their learning, questioning if this is only a responsibility and concern of close relatives to patients with advanced HF.

Furthermore, the study showed that information was conveyed to patients on several occasions where they felt involved. Interestingly, and important for learning was that the healthcare professionals’ use of medical terminology was experienced as a barrier to patients’ and close relatives’ understanding. This problem has been highlighted in several previous studies^37^^-^^40^ and may potentially be overcome by using non-medical terminology.^41^ Nevertheless, in the present study, this is a noticeable finding as patients and close relatives occasionally struggled to build a clear understanding of medical decisions within cross-sectional healthcare. In line with the study by Pedersen and colleagues,^11^ this may indicate that patients with multimorbidity occasionally struggle to comprehend health information. In our study, obtaining information and struggling to comprehend were linked to the individual patient’s or close relatives’ learning processes, and we found that their individual meaning-making processes became important for their learning. Thus, a need exists for healthcare professionals to engage in discussions with patients and close relatives to help them build an understanding and in this way help them manage their life situation with HF and multimorbidity. In this process, patients’ and their close relatives’ preunderstanding of the disease and its treatment will emerge. Unravelling these preunderstandings is important to uncover as this is what new understanding is built on, according to contemporary learning theories.^13^ Sharing knowledge and forming relationships with patients and their close relatives may be time consuming,^42^ which was mentioned by the healthcare professionals in our study. It may be important, though, to allocate time to establish this relationship for various reasons. Firstly, some patients in our study did not have questions because they had years of experience with chronic disease. Secondly, some patients did not perceive themselves as ill although they were affected by multiple diseases. Both of these situations may potentially mask patients' understanding and confusions, potentially influencing their ability to navigate the system and to participate in treatment and care beyond HF, in turn weakening healthcare professionals' capacity to support learning related to treatment and self-management. In our study, it was not always clear to the patients what was relevant to learn, i.e., what was required in a specific situation or which function of a medical device was relevant to understand. Allowing patients and close relatives to express their understandings will allow for feedback and thus facilitate and support learning. Perceiving patients and close relatives as learners would give healthcare professionals a role as facilitators or coaches, which we believe would require pedagogical competencies. In addition, some healthcare professionals felt inadequate when managing patients with comorbidities and were challenged by the complexity of HF treatment. This may potentially explain why some healthcare professionals did not ask about other chronic conditions, risking to indicate that these were of less importance. Knowledge is vital for patient participation;^41^ our findings showed that for appropriately skills among healthcare professionals are of similar importance.

We found that being seen as valuable and being understanding of each individual were preconditions for patients’ involvement and for making decisions within cross-sectional healthcare. This is in accordance with research on PCC,^10^^,^^43^ describing PCC to include aspects such as respect for patients’ preferences, values and expressed needs. Our study showed that for patients and close relatives, involvement in own healthcare within cross-sectional healthcare gave some an opportunity to affect their treatment and care and that this was vital in promoting self-management. Thus, we found that the need to be involved in decision making processes changed over time according to the circumstances and the nature of the choices to be made. Seemingly, it is important to recognise these individual differences to provide more individualised care. Most patients and close relatives in the present study wished to understand and believe in the suggested treatment and wanted to be actively involved in the decision making regarding their treatment course. Fewer wanted the "experts" to choose for them, which has been shown to be the case for other diseases.^37^^,^^44^ Taking up time from busy healthcare professionals made patients in this study feel guilty, which is in line with results from previous research,^37^^, ^^45^^,^^46^ and some experienced treatments as automated procedures. Instead of perceiving care and treatment as individualised, patients felt objectified by the way their treatment and care was organised and conducted. Employing a PCC approach would allow healthcare professionals to get to know the patient and their close relatives beyond his or her biomedical needs, which may contribute to individualising cross-sectional healthcare.

Identification of the GP's role and responsibilities within cross-sectional healthcare seemed to limit a promising PCC approach and thus the experience of continuity in care, which has been described as particularly crucial to patients with HF.^47^^, ^^48^ The present study showed that patients experienced continuity of care in their encounter with the GP when his/her actions were logical and interconnected with prior decisions and when the GP acted in agreement with treatment plans and in accordance with the patients' medical needs and individual situation. This is in line with the Chronic Care Model^18^ and the description of continuity in care by Haggerty and colleagues.^49^ The healthcare professionals in the present study stressed that a lack of continuity in care was caused by longer follow-ups at the hospitals and GPs’ altered roles and practices. Our study showed, however, that some GPs may avoid taking action not to confuse treatment within cross-sectional healthcare. GP inactivity should therefore not always be seen as reluctance to act. Better communication, sharing of information and clear lines of responsibility may be part of future solutions.

Lastly, although close relatives to patients with advanced HF were considered important in terms of providing care, supporting effective self-management and acting as informal caregivers, we found that special attention was not given to their needs and feelings. This has also been reported by previous studies^50^^,^^51^ showing how informal caregivers feel relied upon but not included and showing that their needs to be noticed and recognised by healthcare professionals are not met. According to the healthcare professionals in this study, they perceived having a professional obligation to pay attention to and understand close relatives’ needs. However, they felt that they lacked the time to prioritise this task. Ågren and colleagues^52^ stressed that choosing to priorities this task may potentially promote close relatives’ mental health and improve their capacity to care for the patient. Similar to earlier research^17^^, ^^34^^, ^^50^^, ^^52^ the present study showed that acting as the patient's daily care coordinator cannot be separated from daily life and may occasionally be experienced as burdensome by close relatives and result in a need to air thoughts and frustrations. In the present study, we found that, to a varying degree, close relatives participated in the care of the patient with HF. They wanted, however, a formal invitation to take part and they wanted to be seen. They also wished for professionals to be concerned about their psychological wellbeing, to obtain information and to be intentionally and deliberately involved in the treatment and learning of effective self-management. This would be in line with the core of PCC provision^10^ and with Fitzsimons and colleagues^53^ describing shared decision-making as a process in which the healthcare professionals, the patient and their close relatives jointly agree on treatment and self-management behaviours. Employing a dedicated PCC approach may also resolve the discrepancy in perceptions of "who should take the initiative to invite whom and when", which was observed in our study. We also showed that psychological support for patients with HTx and their close relatives, including children, was missing, which has been acknowledged as a stressful experience by others.^54^ Interestingly, patients in this study perceived that psychological support was their own responsibility as neither they nor their close relatives were offered such support. From the interviews with healthcare professionals, we learned that they felt limited in their options to offer suitable support. The availability of a specialised transplant psychologist for patients and close relatives as a part of the patient pathway for patient undergoing HTx was suggested by patients, and would be in keeping with the PCC approach.^10^ Another promising solution for improving the quality of treatment and care within cross-sectional healthcare was, according to our findings, patients’ and close relatives’ ideas to have a specialised team solely committed to the care and support of close relatives and establishing groups for children to patients/parents with heart disease. We argue that the vulnerability of close relatives in relation to this need should be carefully considered as different kinds of support may be needed.

### Limitations, strengths and methodological discussion

This study has several limitations. First, our findings were derived from the Danish healthcare context and the participants were of Danish origin. Patients with another ethnic background and non-Danish speaking persons would be relevant to include in future research. It would also be relevant to obtain the perspective of their close relatives on cross-sectional healthcare and treatment as they might have additional or different needs for support from healthcare professionals within cross-sectional healthcare. A second limitation was that the study aimed at disclosing the experiences of patients with HF and multimorbidity and hospital-employed healthcare professionals. We realised, however, that some close relatives to patients with HTx and LVAD acted as “gatekeepers” in the patients' care pathway. Thus, it is a limitation that not all close relatives were formally invited to participate in the study. We were in this way confronted with our later findings of a lack of attention to close relatives as we had not invited them in the first place. This was one of the key learning points from our study and it is an important learning point for future research. Importantly, it also seems to require careful considerations in future research that many patients live alone and do not have close relatives but might receive daily support from, e.g., a close friend whom they would like to include. The four close relatives participating in the telephone interviews provided daily support to the patient to a varying extent. Though our study seemed to support patients having a close relative participating, considerations should be made to determine whether both patients and relatives would have revealed other details if the other part had not participated. Splitting up the interviews between patients and relatives may be a solution to this problem. A third limitation was that it was not possible to enrol any GPs in the study; nor were other relevant actors from the municipality considered for inclusion. This should be addressed in future research as their views on cross-sectional healthcare and treatment within this scope of patients and close relatives would be valuable. Fifteen patients were contacted and only one (male) patient declined to participate due to a lack of energy. The majority of participants were male. The underrepresentation of women in clinical research is well-known and has been problematised.^55^ Concerning this study, the male preponderance resulted in a knowledge gap regarding female patients’ views and of the role and needs of their close relatives. Out of nine healthcare professionals, five declined to participate due to a lack of time.

A strength of the study was the use of mixed methods, which supported the establishment of an in-depth understanding of how patients with advanced HF, multimorbidity and CRT, LVAD, or HTx, their close relatives and hospital-employed healthcare professionals experienced cross-sectional healthcare and treatment.

As mentioned earlier, the COVID-19 restrictions forced us to change methods. Thus, the semi-structured interviews with patients and their close relatives were conducted by telephone. Telephone interviews have mainly been used for quantitative surveying,^56^^,^^57^ but have been described as a valid methodological tool that may permit respondents to reveal delicate information more easily.^56^ Sturges and Hanrahan^57^ found no significant difference between telephone and focus group interviews. People are used to communicate both informally and in more formal settings by telephones, which may potentially benefit those who would not have had the time or who had been unable to participate in a face-to-face interview. Moreover, phone interviews allow for more anonymity and privacy. In agreement with Sturges and Hanrahan,^57^ we found that using telephone interviews produced rich and descriptive data. However, at the time we had planned to hold the interviews with healthcare professionals, it was possible to conduct a focus group interview in accordance with our original plan.

## Conclusions

In this study, the experiences with cross-sectional healthcare and treatment were uncovered from the perspectives of patients with advanced heart failure and multimorbidity, close relatives, and specialised hospital-employed healthcare professionals. We argue that cross-sectional healthcare is organised in a way that may influence the patients’ and close relatives’ opportunities to participate in and receive patient-centred care, hereby also influencing their preconditions for adequate self-management and limiting hospital-employed healthcare professionals' provision of patient-centred care. This information may be used in the design of future patient-centred care. Indeed, more patient-centred pathways for patients with advanced HF and multimorbidity may lead to a more detailed, effective, coherent and coordinated experience of cross-sectional healthcare and treatment. We claim, therefore, that improved care delivery models are needed that focus on patients with multiple chronic conditions and on learning. Future care delivery systems should include better IT systems to promote proactive planning, allow for increased involvement of close relatives and for sharing of information across sectors and providers, and they should have a clear distribution of roles and responsibilities between the various stakeholders.

### Practical implications

New complex treatments in advanced HF, the challenges of multimorbidity, the short length of hospital stay, fragmented healthcare provision and the varied and dynamic nature of HF all call for more in-depth knowledge of patients’ and their close relatives’ needs and for a strengthening of patients’ self-management capability. To meet these needs, healthcare professionals’ medical knowledge and competences must be upgraded and their focus on and ability to support patients’ self-management in cross-sectional healthcare setting should be strengthened.

### Main implications for practice, medical education and future research

In this study, we found that cross-sectional healthcare and treatment are characterised by clear distinctions between various disease specialities each of which acts without coordination and with little communication between them. There is a need for development of technological solutions that provide clear communication and enhance the coordination within the healthcare system to design more patient-centred pathways. We also found that close relatives lack attention and support from hospital-employed healthcare professionals. A focus on providing adequate support to close relatives should be mandatory. Future research is warranted to investigate how, when and in what form this support should be provided, and such research needs to be informed by the perspectives of patients and their close relatives. Studies should include male and female patients, with other nationalities than Danish, and their close relatives. In this study, patients and close relatives struggled to understand the language used by professionals during professional encounters. Therefore, healthcare professionals need to express themselves in a more straightforward language. Also, having healthcare professionals understand that professional encounters are also pedagogical encounters would convey occasions for learning. This must be acknowledged and addressed within healthcare to further underpin patient learning and involvement. This would require dedication and allocation of time to establish and engage in pedagogical encounters with patients and close relatives in order to monitor and provide feedback on their learning. We need to acknowledge that we cannot learn for the patient. Rather, we need to go beyond simply supporting patients in remembering and start underpinning the patients’ learning process to ensure that they gain the necessary understanding. Future research should investigate how and to what extent healthcare professionals are capable of using these new pedagogical competences.

The patients and close relatives in this study provided many useful ideas of how to improve the experience of cross-sectional healthcare and treatment. One was an opportunity to speak with a psychologist for both HTx patients and their close relatives as none was offered, not even by request. In future, this would probably be a valuable service.

Another important issue is to consider how to make the findings presented herein part of undergraduate and continuous medical and healthcare education for doctors and nurses alike.

### Acknowledgements

The authors take this opportunity to express our gratitude to the patients, close relatives and hospital-employed healthcare professionals who participated in this study. We also wish to thank the Department of Cardiology at Aarhus University Hospital for supporting the study.

### Conflict of Interest

The authors declare that they have no conflict of interest.

## Supplementary material

Supplementary file 1 Appendix. Experiences with cross-sectional healthcare and treatment in heart failure patients: implications for medical education
