# Supplementary material for: Experiences with cross-sectional healthcare and treatment in heart failure patients: implications for medical education
Source: Int J Med Educ. 2022 Dec 30;13:345–62. doi: 10.5116/ijme.6399.eef4 (PMC10693395; doi:10.5116/ijme.6399.eef4)
Supplement: Supplementary file 1 — Appendix. Experiences with cross-sectional healthcare and treatment in heart failure patients: implications for medical education [file ijme-13-345-S1.pdf]

Appendix

Experiences with cross-sectional healthcare and treatment in heart failure patients: implications for medical education

Main questions	Follow-up questions and probes for clarification
If you look at today's healthcare system for patients with CRT/LVAD/HTx and other chronic diseases. How do you experience this situation and the continuity of care?	What makes healthcare systems and continuity of care difficult and what makes it easier?
How do you experience the encounters within the healthcare system?	Can you describe an exhausting disease pathway? How do you and the patients overcome it?
How do you experience communication within the healthcare system?	Which types of information have you received from healthcare professionals/or provided to patients?
What are your experiences with patients'/healthcare professionals in the management of CRT/LVAD/HTx and other chronic conditions?	How do you/healthcare professionals involve you/the patient in the management of chronic conditions and complex medical treatment? What have you experienced?
Which needs are met, which are not met and why? How can these unmet needs be met?<br>When and how do healthcare professionals/you ask you about other chronic disease?	Does the healthcare professionals/do you ask for your/the patients' experiences, feelings, and needs? Does the healthcare professionals/do you understand and assist you/the patient in managing problems concerning multiple chronic conditions?
How do you experience daily life with your/of patient's condition and other chronic diseases?	Does the healthcare professionals/you ask for your/the patient's ideas and needs when making a treatment plan? Do they/you give you/the patient any choices? Do they/you ask how the plans will affect your/the patient's daily life?<br>How can healthcare professionals/you help or ease the situation for you/the patients?<br>Other patients we have talked to say that they find it difficult to do all the right things, to understand the information and keep a sense of perspective. What are your thoughts on that?<br>How does that affect the family and daily life? What would help to overcome this? What would help to achieve an adequate level of self-management
Patients with multiple chronic conditions and heart disease note that they have many appointments at hospitals, clinics, in general practice, etc. and it is very difficult to stay on top of everything. What are you experiences?	Who helps you to stay on top of all the appointments? Who supports you in doing all the right things?<br>Does the healthcare professionals/do you as healthcare professional ask how many visits you have with other doctors, specialists, surgeons, the general practitioner, etc.? Do you experience continuity in the healthcare service? Why/why not?<br>Do you need support and what would be helpful for you as a patient/healthcare professional? What would not be helpful?
Can you describe your/the general practitioner's role?	I am interested in the experiences you have with your/the patient's general practitioner in managing your/the patient's chronic conditions and complex medical treatment. What are your experiences?<br>What are the barriers or obstacles to achieving this?
In the ideal world, what would you like the healthcare system to provide for patients with CRT/LVAD/HTx and other chronic conditions?<br>– can you think of anything that could be improved?	
	What do you mean?<br>Why do you feel this way?<br>How strongly do you feel then?<br>What did you think then?
